# Pancreatic Necrosis and Gas in the Retroperitoneum: Treatment with Antibiotics Alone

**DOI:** 10.6061/clinics/2017(02)04

**Published:** 2017-02

**Authors:** Roberto Rasslan, Fernando da Costa Ferreira Novo, Marcelo Cristiano Rocha, Alberto Bitran, Manoel de Souza Rocha, Celso de Oliveira Bernini, Samir Rasslan, Edivaldo Massazo Utiyama

**Affiliations:** I Hospital das Clínicas da Faculdade de Medicina da Universidade de São Paulo, Departamento de Cirurgia - Disciplina de Cirurgia Geral e Trauma, São Paulo/SP, Brazil.; II Hospital das Clínicas da Faculdade de Medicina da Universidade de São Paulo, Departamento de Radiologia e Oncologia, São Paulo/SP, Brazil

**Keywords:** Acute Pancreatitis, Necrotizing Pancreatitis, Infected Pancreatic Necrosis, Treatment

## Abstract

**OBJECTIVE:**

To present our experience in the management of patients with infected pancreatic necrosis without drainage.

**METHODS:**

The records of patients with pancreatic necrosis admitted to our facility from 2011 to 2015 were retrospectively reviewed.

**RESULTS:**

We identified 61 patients with pancreatic necrosis. Six patients with pancreatic necrosis and gas in the retroperitoneum were treated exclusively with clinical support without any type of drainage. Only 2 patients had an APACHE II score >8. The first computed tomography scan revealed the presence of gas in 5 patients. The Balthazar computed tomography severity index score was >9 in 5 of the 6 patients. All patients were treated with antibiotics for at least 3 weeks. Blood cultures were positive in only 2 patients. Parenteral nutrition was not used in these patients. The length of hospital stay exceeded three weeks for 5 patients; 3 patients had to be readmitted. A cholecystectomy was performed after necrosis was completely resolved; pancreatitis recurred in 2 patients before the operation. No patients died.

**CONCLUSIONS:**

In selected patients, infected pancreatic necrosis (gas in the retroperitoneum) can be treated without percutaneous drainage or any additional surgical intervention. Intervention procedures should be performed for patients who exhibit clinical and laboratory deterioration.

## INTRODUCTION

Acute pancreatitis is a very prevalent disease worldwide. In the United States alone, 200,000 patients are admitted to the hospital annually due to pancreatitis, and its incidence is increasing 1. Approximately 20% of patients develop severe disease with pancreatic necrosis, which is complicated with the presence of infection in 30% of these patients 2,3. In the last two decades, significant changes have occurred in the treatment of infected pancreatic necrosis. Namely, surgical intervention has been postponed and minimally invasive techniques have been introduced 4-6.

Despite advances in the treatment of acute pancreatitis, the morbidity and mortality of this disease are still very high, particularly when pancreatic necrosis is associated with infection. Failure of one or more organs develops in 40% of patients with pancreatic necrosis, and mortality exceeds 20% when the necrotic area becomes infected 3,5,7.

Although infected pancreatic necrosis can be devastating and can have a poor prognosis, the clinical conditions of patients can be quite variable. Although many patients are critically ill and develop multiple organ dysfunctions, some patients remain clinically healthy 8,9.

Typically, infected pancreatic necrosis must be treated with an invasive procedure. The treatment of infected necrosis can be accomplished by endoscopic, surgical, or percutaneous intervention. In the past, surgical intervention was the first or only option. However, recent studies have advocated the so-called "step-up approach". In this approach, treatment begins with minimally invasive procedures, and operative intervention is performed only when the initial procedure is unsuccessful 2,5,10-13. Percutaneous drainage of infected pancreatic necrotic tissue reduces the need for surgery in up to 35% of cases 11,14.

The timing of intervention in patients with infected pancreatic necrosis has been widely discussed. The current opinion is that later intervention results in a better outcome 6,13. In some situations, the intervention for drainage may be delayed to the point that the patient recovers completely, and no invasive procedure is required. Various studies have demonstrated that selected patients with infected pancreatic necrosis can be successfully managed exclusively by clinical treatment 4,15-17. The objective of this paper is to present our experience in the management of patients with pancreatic necrosis and gas in the retroperitoneum who were treated exclusively with antibiotics and did not require any type of drainage.

## MATERIALS AND METHODS

The General Surgery Service of Hospital das Clínicas of the University of São Paulo School of Medicine maintains a prospective database with detailed records of patients admitted with a diagnosis of severe acute pancreatitis. We retrospectively reviewed the records of all patients admitted to our hospital from January 2011 to June 2015 with acute pancreatitis and infected pancreatic necrosis.

We do not perform fine needle aspiration to determine the presence of infection. The diagnosis of infection of pancreatic necrosis was documented by the presence of gas in the retroperitoneum on computed tomography (CT) or by positive cultures after the drainage of pancreatic collections in patients who maintained clinical or laboratory signs of infection 18.

**Figure 1:**
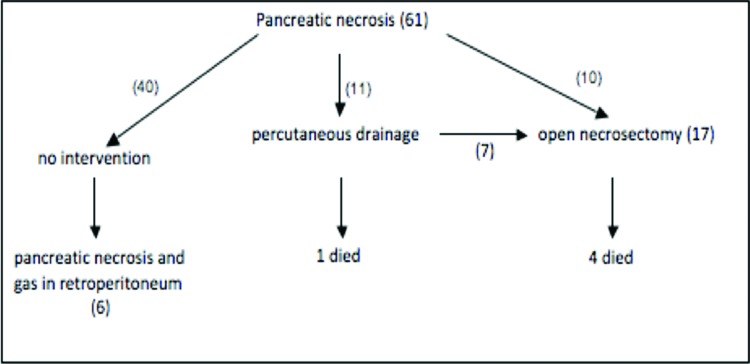
Management and mortality of patients with necrotizing pancreatitis.

We identified 61 consecutive patients with a diagnosis of necrotizing pancreatitis. Forty patients were treated exclusively with clinical support. Eleven patients were initially treated by percutaneous drainage; seven of these patients were subsequently treated with open surgical necrosectomy. Necrosectomy was the first intervention in 10 patients. In the subgroup of 40 patients treated without drainage or surgery, we identified 6 patients with pancreatic necrosis and gas in the retroperitoneum. These 6 patients are the focus of the current study (Figure 1).

The following patient data were reviewed and analyzed: age, gender, aetiology of pancreatitis, length of symptoms before hospital admission, previous visits to the emergency room (ER) at other facilities, Acute and Physiologic and Chronic Health Evaluation II (APACHE II) score on admission, length of hospital stay, length of readmission, number of CT scans performed, modified Balthazar CT severity index score 19, the length of antibiotic therapy, positive blood cultures, the need for enteral feeding, white cell count, and C-reactive protein (CRP) (Tables 1 and 2).

## RESULTS

Tables 1 and 2 summarize the main characteristics of the 6 patients. In one patient, the aetiology of pancreatitis was alcoholism. In all the other patients, pancreatitis occurred secondary to gallstones. One of these 5 patients also had intraductal mucinous neoplasms with calcification in the pancreatic head.

Only one patient was treated by our service beginning from the onset of symptoms. The other 5 patients were previously treated at other facilities and were admitted to our service more than 2 weeks after the beginning of the disease.

All patients were clinically healthy at the time without any organ dysfunction. Only two patients had an APACHE II score of >8. The maximum CRP levels were 372 mg/L in one patient and >150 mg/L in 3 patients. Leucocytosis was present in only one patient.

**Figure 2:**
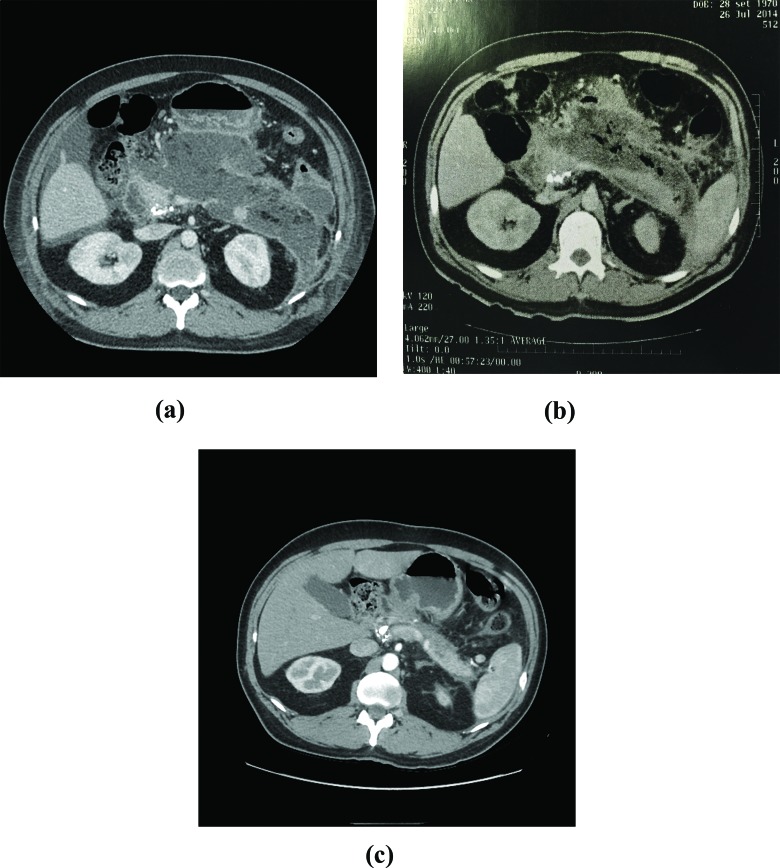
CT scan, case 5. **A:** First exam, 10 days after the onset of symptoms: extensive necrosis of the body and tail of the pancreas; calcification in the pancreatic head, suggesting the presence of intraductal neoplasia. **B:** After 30 days of follow-up (increase in CRP and fever): gas in the full extent of the area of pancreatic necrosis. **C:** 5 months after hospital discharge: no pancreatic necrosis or collection.

**Figure 3:**
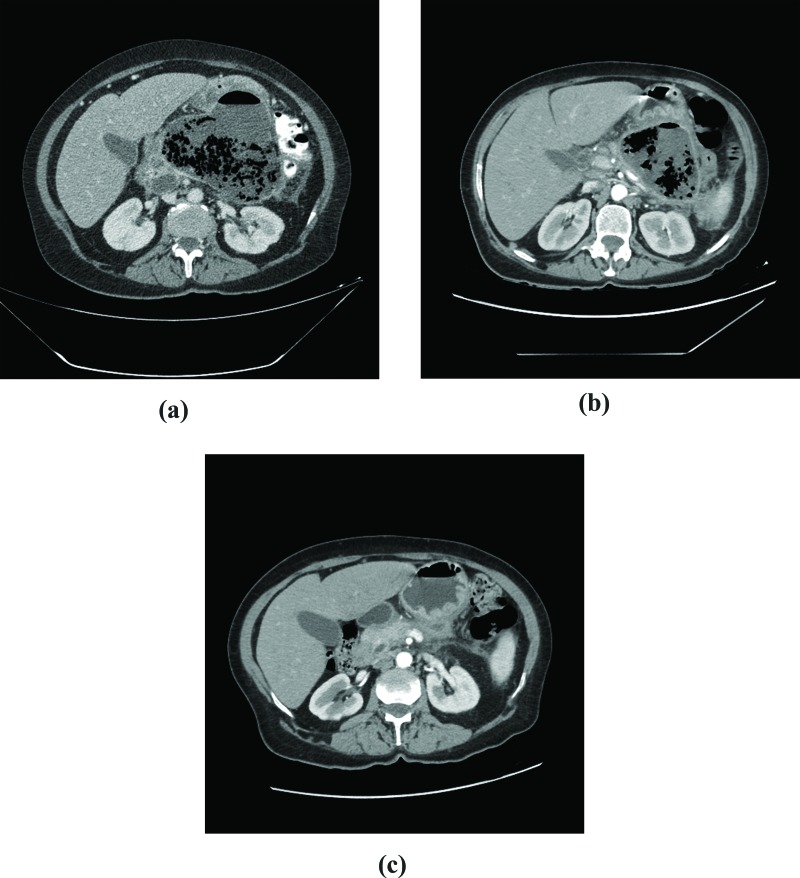
CT scan, case 4. **A:** First exam, 3 weeks after the onset of symptoms: extensive pancreatic necrosis with a large amount of gas in the body and tail of the pancreas. **B:** After 60 days of follow-up: a small decrease in the collection of gas is noted. **C:** 4 months after hospital discharge: atrophy of pancreatic parenchyma.

**Figure 4:**
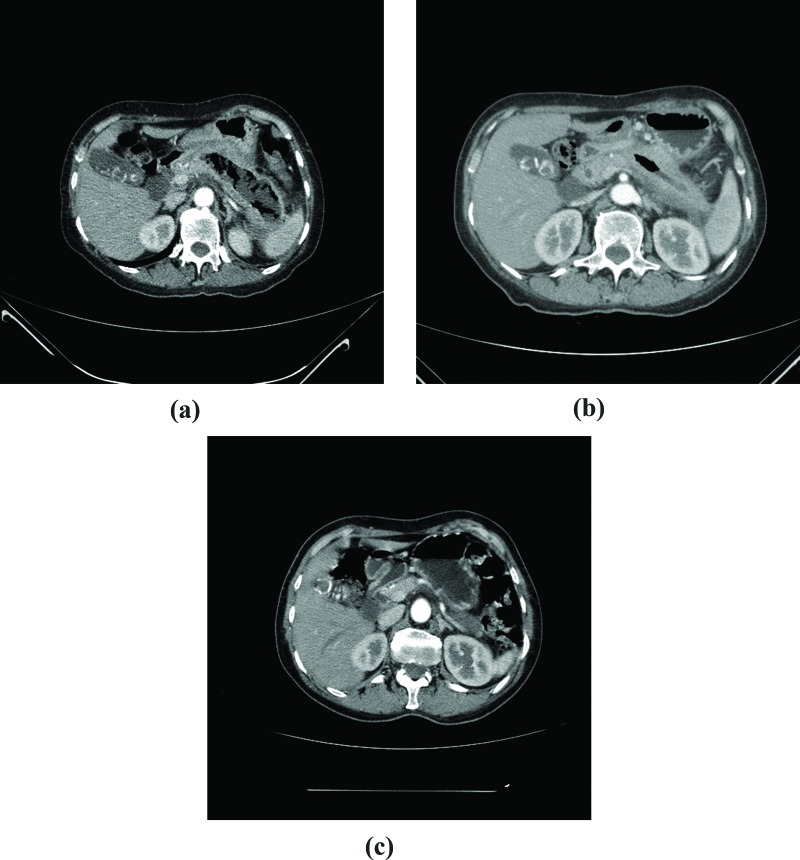
CT scan, case 3 **A:** First exam, 3 weeks after the onset of symptoms: pancreatic necrosis with gas throughout the body and the tail of the pancreas. **B:** After 50 days of follow-up: significant necrosis and gas are still present. **C:** 3 months after hospital discharge: no pancreatic necrosis or collection.

The first CT performed in our service revealed gas in all but 1 patient (case 5) (Figure 2). All patients underwent at least 3 CT scans (Figures 2, 3 and 4). Balthazar’s CT severity index score was 9 in 5 patients (Table 2).

All patients were treated with antibiotics due to the presence of gas in the retroperitoneum. The initial antibiotic therapy consisted of ciprofloxacin plus metronidazole in all patients. In 1 patient, this scheme was changed to imipenem due to the presence of a persistent fever. All patients received antibiotics for at least 3 weeks. Only 2 patients had positive blood cultures.

No patient received parenteral nutrition. Four patients were treated with enteral nutrition through tubes placed endoscopically at a location beyond the angle of Treitz.

The length of the hospital stay exceeded 3 weeks for all (23-36 days) but 1 patient (case 6). Three patients were readmitted due to persistent vomiting and fever. All readmitted patients were again treated with antibiotics. Two patients received nutrition through enteral tubes.

A cholecystectomy was performed in the 5 patients diagnosed with cholelithiasis after control CT scans revealed complete recovery of pancreatic necrosis. Two patients (cases 4 and 5) experienced recurrence of pancreatitis with infected pancreatic necrosis approximately six months after hospital discharge. The patients were waiting to receive a cholecystectomy at that time. Both patients exhibited an uneventful recovery from this new episode and have subsequently received surgery. No deaths occurred in this series.

## DISCUSSION

This study analyzed six patients with infected pancreatic necrosis confirmed by the presence of gas in the retroperitoneum who were treated exclusively with antibiotics. The spectrum of clinical presentations of patients with acute pancreatitis can vary widely and can be disproportionate to CT findings. Therefore, treatment should be individualized and dictated mainly by the clinical condition of the patient.

In our study, only 2 patients had an APACHE score >8, whereas the CT index score, as proposed by Balthazar 19, was 9 in 5 patients. The systemic conditions do not always reflect the severity of pancreatic and peripancreatic compromise. Lankisch et al. 20 demonstrated that the APACHE II score was not the best tool to evaluate the presence and severity of pancreatic necrosis. The APACHE II score has a sensitivity of 36% and a specificity of 72%. Our study also demonstrated that only 2 patients had an APACHE II score >8 despite extensive pancreatic necrosis with gas.

In 2012, Dellinger et al. 8 proposed a new classification of pancreatitis that takes into account both systemic and local conditions. The Atlanta classification includes only systemic conditions and was revised in 2013 21. The new classification includes moderate acute pancreatitis, a condition in which the patient has pancreatic or peripancreatic necrosis but no organ dysfunction 9.

The classic treatment of infected pancreatic necrosis is operative intervention with necrosectomy. This invasive procedure has a high incidence of complications and mortality (13-26%), even in specialized services 4,6,22. Therefore, minimally invasive interventions, mainly percutaneous and endoscopic drainage, have been proposed 2. The pancreatitis, necrosectomy versus step up approach study (PANTER) 5,10,23,24 compared patients randomized to receive open necrosectomy or minimally invasive procedures (endoscopic or percutaneous drainage) as the first intervention. The group that was initially treated with minimally invasive drainage exhibited a reduced incidence of complications and multiple organ failure. Hollemans et al. 11 demonstrated that percutaneous treatment was the only intervention necessary for many patients initially treated by this "step-up approach". The need for operative treatment was eliminated in 35% of their patients. These data are similar to the results of some retrospective studies that have demonstrated that percutaneous drainage obviates the need for surgery in up to half of all cases 25,26.

Due to increased use of the "step-up approach", open necrosectomy has been performed less frequently. However, Madenci et al. 27 reported a mortality rate of only 8.8% in patients who underwent pancreatic open necrosectomy, but only 17% of the patients underwent percutaneous drainage before the operation.

When used as the only treatment or along with a minimally invasive procedure when feasible, medicinal therapy may avoid the complications associated with open necrosectomy, such as new onset or worsening of multiple organ dysfunction, bleeding, fistulae, pancreatic insufficiency and incisional hernia 4,27,28.

In 1996, Dubner et al. 17 first reported the treatment of 3 patients with infected pancreatic necrosis without invasive interventions. The infection was diagnosed by fine needle aspiration, and patients were treated with antibiotics alone.

In 2005, Runzi et al. 16 published a study of 28 patients with infected pancreatic necrosis. Sixteen of these patients received no operative treatment. Six patients recovered uneventfully. The other patients exhibited multiple organ failure. The mortality rate was 12.5%. One drawback of the aforementioned study was that only 3 patients underwent percutaneous drainage, although 10 patients had multiple organ dysfunction.

Lee et al. 15 also described non-operative treatment for infected pancreatic necrosis in 31 patients. In their study, 74% of patients were initially treated with minimally invasive drainage. Four of these patients underwent open necrosectomy after percutaneous or endoscopic drainage. Eight patients (25%) were treated exclusively with antibiotics, and only 1 patient died.

We believe that early treatment with antibiotics must be the initial choice for all patients with infected pancreatic necrosis. If the patient presents with signs of clinical worsening, a minimally invasive procedure should be promptly considered.

After the publication of the PANTER study 5,10,23, the indication of percutaneous drainage became more liberal, but some patients can be successfully treated exclusively with antibiotics 4,15-17.

Percutaneous drainage and open necrosectomy are both associated with a high incidence of pancreatic fistulae 27, and in some series, the incidence is up to 50%. This finding may justify a tendency to postpone the use of invasive treatment in patients with infected pancreatic necrosis 29.

Regarding the treatment of infected pancreatic necrosis, the timing of the intervention may have a significant impact in the outcome 13,30. Götzinger et al. 6 observed a mortality rate of 46% in patients who received surgery during the first 3 weeks after the diagnosis of pancreatitis *versus* a mortality rate of 25% in patients who underwent surgery after this period.

We believe that invasive treatment of infected pancreatic necrosis should be delayed as long as possible. Drainage should be conducted only if clinical and laboratory findings indicate that the patient is deteriorating despite the use of appropriate antibiotic therapy. Currently, in our service, antibiotics are only used for patients with documented or presumed infected necrosis. We no longer use antibiotics for infection prevention, even when extensive necrosis is observed 31,32. The length of antibiotic therapy remains an open issue. Runzi et al. 16 administered antibiotics for at least 8 weeks. We believe that the treatment of infected necrosis requires prolonged antibiotic therapy regardless of the use of interventional procedures. We advocate that antibiotic use should be continued for at least 3 weeks.

The diagnosis of infection in patients with pancreatic necrosis remains a substantial challenge. In the past, the pressure to document necrosis infection was higher because physicians believed that surgical treatment should be immediately instituted once an infection was diagnosed 13. The presence of gas in the retroperitoneal collection is considered to indicate the presence of an infection. Infection can also be diagnosed by the presence of microorganisms in cultures of material collected from the necrotic area by fine needle aspiration or during drainage or open surgery. However, the sensitivity of fine needle aspiration is only 50 to 70%. The sensitivity may be low due to the use of preventive antibiotics in some cases 2,30. We consider fine needle aspiration to be an invasive procedure that involves the transfer of the patient from the intensive care unit to the radiology department. In patients who maintain organ dysfunction despite the administration of appropriate therapy, the necrotic area should be presumed to be infected. It is believed that many patients with pancreatic necrosis who improve after antibiotic therapy have infected pancreatic necrosis that is not documented 2,18,33.

Regardless of the type of therapy chosen, the treatment of infected pancreatic necrosis requires a prolonged hospital stay 5,15,27. In patients treated with antibiotics alone, the length of the hospital stay is also very long and comparable to that of patients who undergo minimally invasive or surgical procedures 5,15. Van Santvoort et al. 5 reported no difference in the length of hospital stay in patients who received the “step-up approach” compared to patients in whom surgical treatment was the first intervention. In our study, the length of the hospital stay exceeded three weeks for all patients. In addition, 3 patients required early hospital readmission.

Extensive pancreatic necrosis, particularly when an infection is present, increases the risk of bleeding from large splanchnic vessels. This finding explains why many surgeons feel uncomfortable if they do not intervene in cases of infected necrosis. However, bleeding usually stops spontaneously due to the rigidity of the local inflammatory tissues 29. In our series of 61 patients, 1 patient with infected pancreatic necrosis who was initially managed only with antibiotics was diagnosed as having a colon perforation secondary to the thrombosis of colic vessels 5 weeks after treatment initiation. This case emphasizes the need for close and very careful monitoring of patients, possibly for long periods of time. Some may argue that this complication could have been avoided if drainage had been performed earlier during the course of pancreatitis.

This study shows that, in selected cases, infected pancreatic necrosis with gas in the retroperitoneum requires neither immediate percutaneous drainage nor surgical intervention. These procedures should be reserved for patients who develop signs of clinical and laboratory deterioration. Some patients can be safely treated with antibiotics alone.

## AUTHOR CONTRIBUTIONS

All authors involved in this study agreed with the submission. Rasslan R and Novo FC were responsible for the study conception and design, data acquisition, analysis and interpretation. Rocha MC, Bitran A and Rocha MS were involved in the acquisition and the analysis of data. Utiyama EM, Bernini CO and Rasslan S were responsible for the manuscript drafting and critical revision for important intellectual content.

## Figures and Tables

**Table 1:** Demographic and clinical characterization of patients.

Patient	Age	Gender	Aetiology	Length of Symptoms(days)	Previous visits to the ER	Length of stay (days)	Readmissionafter discharge/length of stay (days)
1	69	M	Cholelithiasis	21	+	24	5/30
2	17	M	Cholelithiasis	14	+	32	-
3	58	F	Cholelithiasis	20	+	5	-
4	69	F	Cholelithiasis	20	+	36	-
5	40	M	Cholelithiasis	10	-	23	15/22
6	40	M	Alcohol	23	+	12	8/5

**Table 2:** Laboratory and radiological data and treatment.

Patient	APACHE II score	CRP (mg/L)	White blood cells (x10^3^)	Number of CTs	Balthazar CT score	Blood culture	Enteral tube feeding
1	6	117	10.62	6	9	Streptococcus anginosus	+
2	5	159	20.12	5	9	Micrococcus	+
3	5	30	9.47	3	9	-	-
4	8	198	7.86	4	9	-	+
5	10	372	10.88	4	9	-	+
6	0	78	12.00	4	5	-	-
